# Genetically Determined Physical Activity and Its Association with Circulating Blood Cells

**DOI:** 10.3390/genes10110908

**Published:** 2019-11-07

**Authors:** Femke M. Prins, M. Abdullah Said, Yordi J. van de Vegte, Niek Verweij, Hilde E. Groot, Pim van der Harst

**Affiliations:** Department of Cardiology, University of Groningen, University Medical Center Groningen, 9713 GZ Groningen, The Netherlands; f.m.prins@umcg.nl (F.M.P.); m.a.said@umcg.nl (M.A.S.); y.j.van.de.vegte@umcg.nl (Y.J.v.d.V.); n.verweij@umcg.nl (N.V.); h.e.groot@umcg.nl (H.E.G.)

**Keywords:** physical activity, blood cell counts, single nucleotide polymorphisms, inflammation

## Abstract

Lower levels of physical activity (PA) have been associated with increased risk of cardiovascular disease. Worldwide, there is a shift towards a lifestyle with less PA, posing a serious threat to public health. One of the suggested mechanisms behind the association between PA and disease development is through systemic inflammation, in which circulating blood cells play a pivotal role. In this study we investigated the relationship between genetically determined PA and circulating blood cells. We used 68 single nucleotide polymorphisms associated with objectively measured PA levels to perform a Mendelian randomization analysis on circulating blood cells in 222,645 participants of the UK Biobank. For inverse variance fixed effects Mendelian randomization analyses, *p* < 1.85 × 10^−3^ (Bonferroni-adjusted *p*-value of 0.05/27 tests) was considered statistically significant. Genetically determined increased PA was associated with decreased lymphocytes (β = –0.03, SE = 0.008, *p* = 1.35 × 10^−3^) and decreased eosinophils (β = –0.008, SE = 0.002, *p* = 1.36 × 10^−3^). Although further mechanistic studies are warranted, these findings suggest increased physical activity is associated with an improved inflammatory state with fewer lymphocytes and eosinophils.

## 1. Introduction

Reduced physical activity (PA) poses a serious threat for public health. Accumulating evidence shows that lower levels of PA are associated with an increased risk of cardiovascular disease (CVD) and all-cause mortality [1,2,3]. Although the World Health Organization (WHO) recommends at least 150 minutes of moderate-intensity aerobic PA throughout the week, the proportion of Europeans reported to not meet these recommendations has increased in recent years to 46% [4,5]. This trend is not limited to Europeans but is occurring worldwide [6]. Although health care cost estimates related to physical inactivity vary across studies (e.g., 2.4–11.1% of the healthcare expenditure in the United States of America), it is generally believed that physical inactivity is a costly pandemic and associated with a substantial disease burden [7]. 

However, the exact mechanisms underlying the associations of PA the development of disease are incompletely understood. It has been suggested that systemic inflammation plays a pivotal role in the association between PA and CVD, possibly through changes in circulating (inflammatory) blood cells [8,9]. It is therefore important to investigate whether the effects of PA on CVD risk could be linked through changes in circulating blood cells. However, the association between PA activity and circulating blood cells has only been investigated using traditional observational analyses, which are prone to suffering from confounding effects [10,11]. PA is determined by both genetic and environmental factors [6,12]. A recent genome-wide association study (GWAS) using objectively measured data from wrist-worn accelerometers of PA in a large sub-cohort of the UK Biobank identified newly associated single nucleotide polymorphisms (SNPs) and studied whether activity might contribute causally to disease outcomes [12]. In this study, we aimed to investigate the relationship between genetically determined PA levels, based on the previously reported SNPs, and circulating blood cells using a Mendelian randomization (MR) strategy to minimize confounding effects. We hypothesize a genetically determined higher level of PA is associated with a lower inflammatory state with fewer circulating inflammatory blood cells.

## 2. Material and Methods

### 2.1. UK Biobank Participants

The UK Biobank study design and population have been described in detail elsewhere [13]. In brief, the UK Biobank is a large community-based prospective study in the United Kingdom that recruited over 500,000 participants aged 40–69 years aiming to improve the prevention, diagnosis, and treatment of a plethora of diseases. All participants gave informed consent for the study [13]. At the baseline visit, vital signs and biological samples were collected, together with data of self-completed questionnaires, interviews, and physical measurements. The present study was conducted under application number 12,006 of the UK Biobank resource. 

### 2.2. Genotyping and Imputation

**Figure 1:**
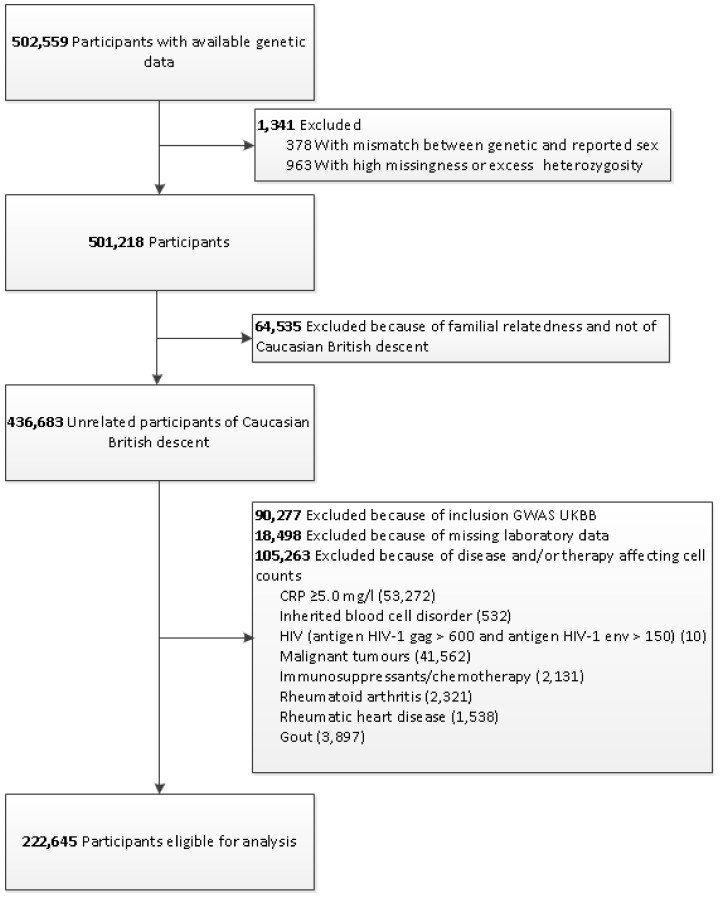
Flowchart for the selection of the analyzed study sample from the UK Biobank Study. GWAS: Genome-wide association study; UKBB: UK Biobank; CRP: C-reactive protein; HIV: human immunodeficiency virus.

The genotyping process and arrays used in the UK Biobank study have been described elsewhere in more detail [14]. Briefly, participants were genotyped using the custom UK Biobank lung exome variant evaluation axiom (Affymetrix: Santa Clara, CA, United States; n = 49,949), which includes 807,411 SNPs or the custom UK Biobank axiom array (Affymetrix; n = 452,713), which includes 820,967 SNPs [13]. The arrays have insertion and deletion markers with more than 95% common content [14,15]. Imputed genotype data were provided by UK Biobank, based on merged UK10K and 1000 Genomes phase 3 panels [16]. Figure 1 shows a flowchart of the study sample selection and is further described below. Participants were excluded if there was no genetic data available or if there was a mismatch between genetic and reported sex (n = 378). Furthermore, participants with high missingness or excess heterozygosity were excluded (n = 963). Participants with familial relatedness or who were not of white British descent were excluded as well (n = 64,535). In addition, participants included in the GWAS on physical activity (n = 90,277) [12] and participants without lab measurements were excluded (n = 18,498). Lastly, participants with diseases or medication affecting the immune response were excluded as well (n = 105,263). We created a set of 222,645 individuals for the present analyses. 

For the definitions of diseases, we used hospital episode statistics data in combination with self-reported diagnoses and medication, as described previously [17]. Further information on the definitions of diseases is presented in Supplementary Table S1.

### 2.3. Single Nucleotide Polymorphisms

For our analyses between genetically determined PA and circulating blood cells, we used a set of 68 SNPs identified in the GWAS on physical activity by Doherty et al. [12]. Similar to Doherty et al., we used 68 SNPs that were associated with physical activity with *p <* 5 × 10^−6^ (Doherty et al., Supplementary Figure S8) to explain more phenotypic variance than the three SNPs at *p <* 5 × 10^−8^. Supplementary Table S2 contains a detailed list of the extracted SNPs. SNPs associated with sleep duration in the study by Doherty et al. were not present in this list of SNPs.

### 2.4. Statistical Analyses

Normally distributed continuous variables were summarized as mean ± standard deviation (SD) and skewed variables as median and interquartile range (IQR). Linear regression analyses were performed to assess the association between PA and blood cell counts. Regression analyses between SNPs and circulating cells were adjusted for age at the baseline visit, sex, genotyping chip, and the first 30 principal components provided by UK Biobank (to adjust for population structure). Because the PA SNPs were identified in a GWAS on objectively measured PA, the associations between the SNPs with self-reported PA were not tested as these were considered separate entities. Linear regression analyses were performed using Stata 15 (StataCorp, College Station, TX, United States). 

The association between genetically determined increased physical activity with outcomes was first assessed using a fixed-effects inverse-variance weighted (IVW) meta-analysis method, combining the Mendelian randomization (MR) estimates for each SNP with the outcome. To adjust for multiple testing, we applied a Bonferroni correction (significance level divided by number of independent tests) and considered a two-sided *p*-value of less than 0.05/27 = 1.85 × 10^−3^ as statistically significant for the main analyses using the MR-IVW fixed effects model. For the MR-IVW effects model to be valid, the assumption of absence of pleiotropy needs to be fulfilled. Pleiotropy occurs when genetic variants associated with the exposure of interest, exert their effect on the same outcome through multiple pathways. Heterogeneity tests are an easy way to evaluate possible pleiotropy, since low heterogeneity indicates that estimates between the genetic variants’ association with the outcome should vary by chance only, which is only possible in case of absence of pleiotropic effects. The Rücker framework was adopted to differentiate between preferred models. In case Cochran’s Q in the MR-IVW fixed effects analyses was significant (*p* < 0.05), suggesting heterogeneity and thus non-random error, the MR-IVW random effects model was adopted. Rücker’s Q was calculated to evaluate the heterogeneity within the MR-Egger analyses [18]. We then calculated whether the Rücker’s Q’ statistic was different (*p <* 0.05) from Cochran’s Q statistic (Q-Q’) [18]. A statistically significant difference indicates the MR-Egger test to be the best approach in case Q-Q’ is large and positive [18]. MR-Egger assumes pleiotropic effects of the SNPs on the outcome are independent of their association with PA, and therefore allows for a non-zero intercept [11]. For this, the MR-Egger must not violate the InSIDE assumption, which assumes the association of the SNPs with the exposure are independent of their direct pleiotropic effects on the outcome. The MR-Egger provides additional information on pleiotropy as a non-significant different intercept from 0 (*p >* 0.05) indicates evidence for absent pleiotropic bias. MR-Steiger filtering was performed to remove variants with stronger associations (R^2^) with the outcome than the exposure [18]. Beta values (β) and standard errors (SE) are provided for the MR outcomes. Lastly, we performed a weighted median analysis, which allows up to 50% of the information from variants to violate the MR assumptions [19]. For sensitivity analyses, we adopted a *p*-value of <0.05. 

MR analyses were performed using the R (version 3.5.1) and the package TwoSampleMR version 0.4.22 (https://mrcieu.github.io/TwoSampleMR/).

## 3. Results

### 3.1. Population Characteristics

Baseline characteristics are provided in Table 1. Of the 222,645 participants included in the UK-Biobank, 105,970 (47.6%) were male, and the mean age was 56 ± 8 years. The population was slightly overweight (body mass index ≥25 kg/m^2^) with a mean body mass index of 27.0 kg/m^2^. More than half of the population never smoked or smoked <100 cigarettes. On average, participants spent 4.88 (inter quartile range (IQR): 1.5–11.3) hours per week doing moderate PA and 0.75 (IQR 0.0–2.7) hours per week doing vigorous PA, based on self-reported data using questionnaires. 

### 3.2. Genetically Determined Physical Activity and Circulating Blood Cells 

**Figure 2:**
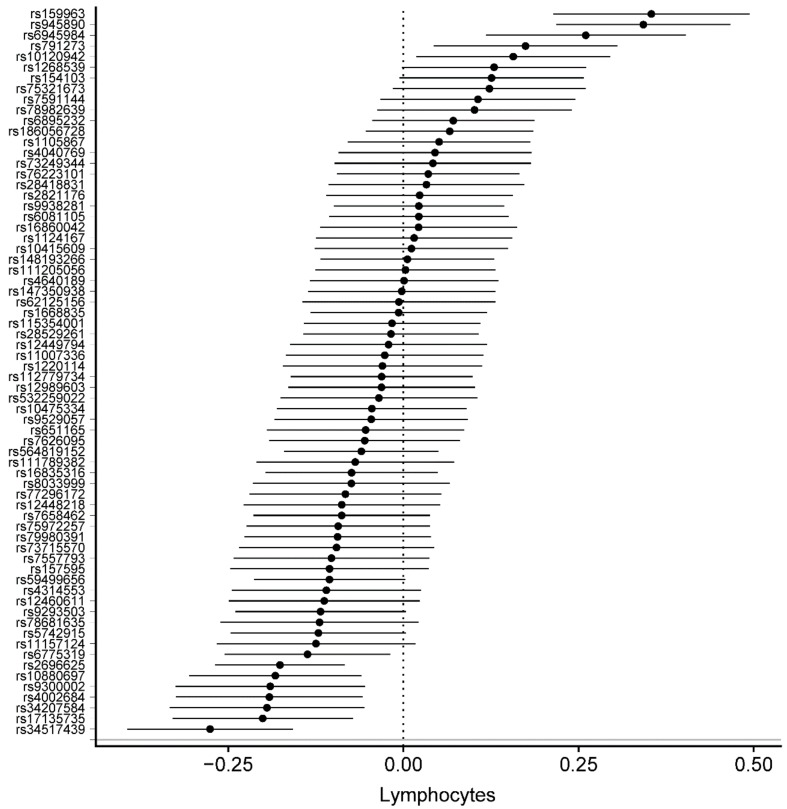
Forest plot of physical activity single nucleotide polymorphisms on lymphocyte levels.

**Figure 3:**
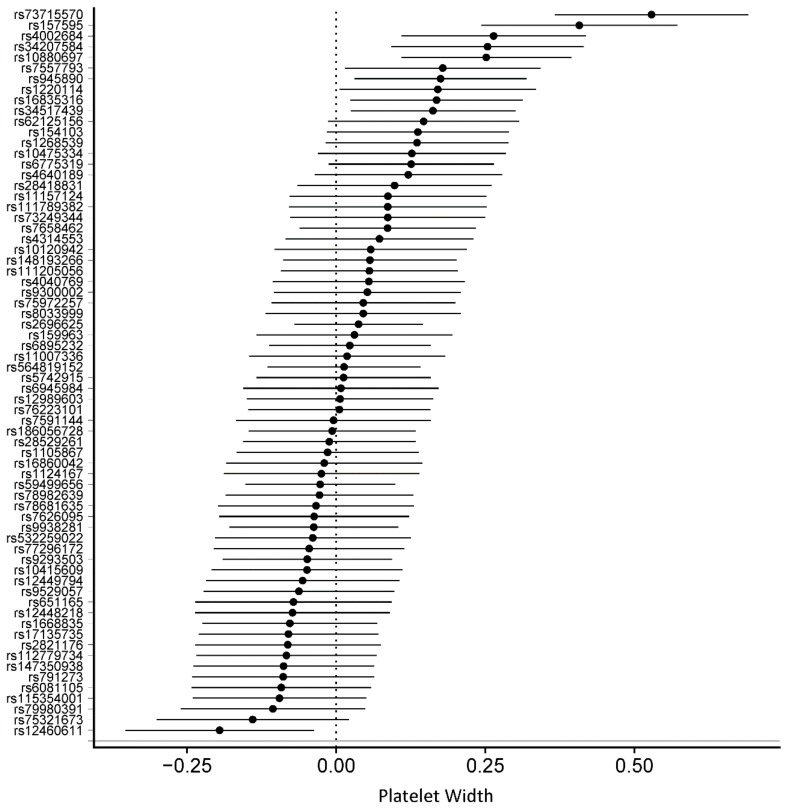
Forest plot of physical activity single nucleotide polymorphisms on platelet width.

**Figure 4:**
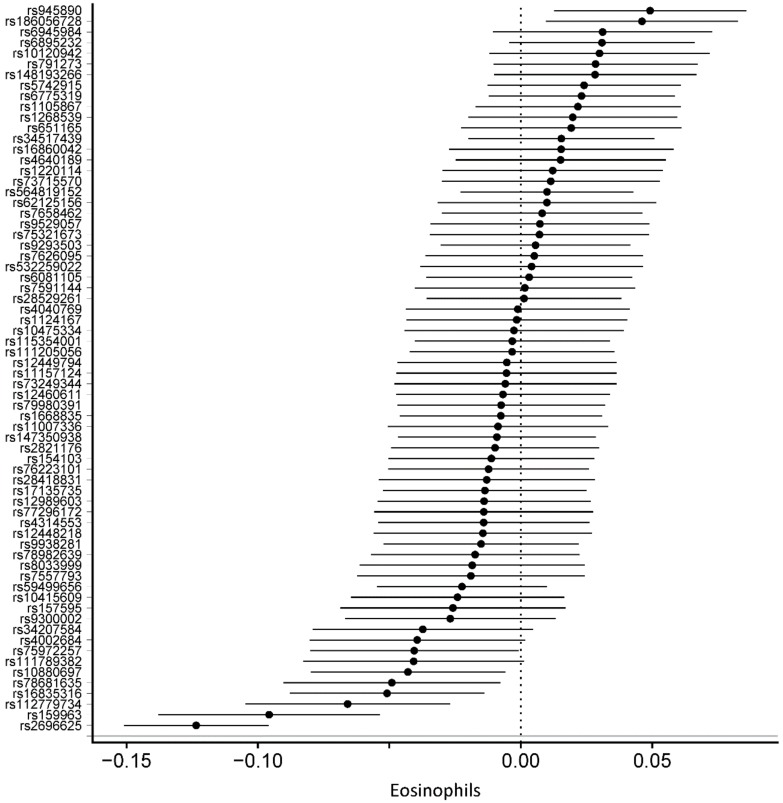
Forest plot of physical activity single nucleotide polymorphisms on eosinophil level.

Detailed information on the SNPs and their estimates on circulating blood cells is provided in Supplementary Table S3. The association between all 68 SNPs associated with PA with circulating blood cells was assessed using MR analyses. In MR-IVW fixed-effects analyses, genetically determined increased duration of PA was associated with decreased lymphocytes (β = –0.026, SE = 0.008, *p* = 1.35 × 10^−3^), decreased eosinophils (β = –0.008, SE = 0.002, *p* = 1.36 × 10^−3^), and increased platelet distribution width (β = 0.04, SE = 0.009, *p* = 9.07 × 10^−5^) (Table 2). Figure 2, Figure 3 and Figure 4 displays the individual SNP forest plots for these three outcomes. Supplementary Figure S1–S3 displays the corresponding scatter plots. PA was not associated with any of the other outcomes in the MR-IVW fixed-effects analyses (Table 2), which will therefore not be discussed any further. 

Cochran’s Q of the association between PA and lymphocytes (Q = 160, DF = 67, *p* = 6.50 × 10^−10^) and eosinophils (Q = 180, DF = 67, *p* = 6.10 × 10^−12^) indicated the MR-random effects model to be the preferred approach. Using this approach, the association with eosinophils remained significant (β = −0.0078, SE = 0.009, *p* = 0.049), but the association with lymphocytes was attenuated (β = −0.026, SE = 0.014, *p* = 0.072). However, the loss of statistical significance was probably attributable to the larger standard error due to loss of power in the random effects model. In the weighted median MR analyses, the associations between PA and leukocytes (β = −0.030, SE = 0.0134, *p* = 0.022) and eosinophil count (β = −0.0036, SE = 0.0036, *p* = 0.3229) were lost, of which the latter could be attributed to wider standard errors. MR-Steiger filtering indicated all SNPs were more strongly associated with the PA behavior than with lymphocytes and eosinophils. The MR Egger intercept indicated little evidence for pleiotropy for the analyses on lymphocytes and eosinophils. 

As an additional analysis, we investigated whether the relationship between genetically determined PA with circulating lymphocytes or eosinophils differed between levels of self-reported PA. However, genetically determined PA was not significantly associated with either lymphocytes or eosinophils amongst individuals with no (n = 16,307), only moderate (n = 52,273), or only vigorous (n = 4536) self-reported PA.

In the heterogeneity analyses, Q-Q’ was statistically significant for platelet distribution width (Q = 160 DF = −67 *p* = 1.80 × 10^−3^), indicating the MR-Egger analysis to be the best approach. Using this approach, the association between genetically determined PA with platelet distribution width was reversed in effect and no longer significant (β −0.05, SE 0.04, *p* = 0.26). Furthermore, we performed a look-up in MR-Base to explore whether the 68 genetic variants were associated with other traits than PA. This information can be found in Supplementary Table S5.

## 4. Discussion

In the present study, we provide evidence for the association between genetically determined PA and circulating blood cells. Genetically determined increased duration of PA was cautiously associated with decreased lymphocytes and decreased eosinophils, suggesting increased levels of PA may improve the inflammatory state. This is in line with our hypothesis.

The present study is the first to report associations between genetically determined PA and circulating blood cells. The association between genetically determined increased PA and decreased lymphocyte levels is partly in line with previous research which studied the association between PA and total leukocyte count [8]. In 4,857 individuals with a mean age of 43 ± 1 year and 43% females, participating in the National Health and Nutrition Examination Survey, the association between increased PA and a decreased leukocyte count has been observed, suggesting that active individuals might maintain a lower inflammatory state and might be less prone to future chronic disease development [8]. We did not assess the correlations between self-reported PA and circulating blood cells, and this study can therefore not be directly compared with these previous studies. However, our study is of additive value, since we were able to study the association between PA and a broader range of cell types (i.e., monocytes, lymphocytes, neutrophils, eosinophils, and basophils) instead of total leukocyte count solely. We did not observe an association between genetically determined PA and total leukocyte count, but we did observe an association between increased genetically determined PA and decreased lymphocytes levels in the MR-IVW analyses. For a long period, a decrease in lymphocytes has been considered as a suppression of the human immune system and therefore as detrimental [20]. However, recent evidence indicates that this reduction in peripheral blood does not reflect immune suppression, but represents a heightened state of immune surveillance and immune regulation, which is driven by a transfer of cells to peripheral tissues, such as the gut or the lungs [21]. Within the cardiovascular field, T-lymphocytes are known to stimulate macrophages expressing collagen-degrading enzymes and thereby increasing the risk of plaque rupture. Lower lymphocyte levels in the blood stream, might therefore also be beneficial for the risk of CVD [22,23]. The association between lymphocyte count and PA in our study was lost in the sensitivity analyses, although the strength of the effect remained similar. This loss of association may be due to a larger standard error reducing the statistical power. Similarly, no association was observed between genetically determined PA and lymphocytes or eosinophils across levels of self-reported PA, which is likely due to the small sample sizes in these groups. Further MR studies using variants which explain a larger variance in PA and also in larger groups of self-reported PA levels are warranted to further investigate these associations. 

There is limited data on the association between PA and eosinophils. Earlier research in 11 athletes running an ultramarathon (90 kilometers), showed a non-allergic activation of eosinophils, reflected by an increase of eosinophil cationic protein [24]. Our study population of community dwelling middle-aged men and women substantially differed from those athletes (e.g., age, ethnicity, and athletic condition), and therefore, it is not possible to formulate any hypotheses or to draw any comparisons [24]. Recently, in 5287 patients who underwent coronary angiography, a negative association between peripheral eosinophil count and the severity of coronary artery disease (CAD) has been observed [25]. Furthermore, previous studies have suggested that eosinophils play a key role in the initiation, progression, and rupture of thrombotic plaques, which was confirmed by tissue samples obtained through thrombus aspiration in patients with myocardial infarction [26,27]. In these samples, a large amount of eosinophils was present [26]. However, these findings (low peripheral eosinophil count associated with increased CAD severity and high eosinophil counts observed in atherosclerotic plaques) were observed in CAD patients, whereas our study was performed in a general population of which only 3.7% had a medical history of CAD at baseline. The present findings indicate increased PA is associated with decreased eosinophil levels. Possibly, less PA leading to higher eosinophil levels could play a role in the development of plaques eventually leading to CAD. In this case, increased PA leading to lower eosinophil levels might be protective for plaques and CAD. Further research is needed to investigate this hypothesis.

Contrary to previous cross-sectional observational studies, we did not observe associations between genetically determined PA and red blood cell indices [28,29,30]. This might imply that changes in red blood cells during or after PA are bystanders instead of a consequence of PA, although mechanistic studies are necessary to unravel these associations.

Of interest is the specific function of three leading genetic polymorphisms rs564819152, rs2696625, and rs59499656, which may provide more insights in the mechanisms underlying genetically determined PA and circulating immune cells. rs564819152 is located near the SKIDA1 gene. This gene is located on chromosome 10 and encodes the Ski-Dach domain-containing protein 1, which is associated with different types of cancer [31,32]. Furthermore, this gene was found to be associated with lung function [33]. These functions might affect the relation between PA and blood cells, although our heterogeneity tests did not indicate pleiotropy of the genetic variants. 

rs2696625 and rss59499656 are located near the genes KANSL1-AS1 and SYT4, respectively. KANSL1-AS1 has been described in the context of Alzheimer’s and Parkinson’s diseases [34]. SYT4 (synaptotagmin 4) is a protein coding gene that has been associated with various traits, i.e., body mass index, lung function, and body fat percentage [33,35,36]. As obesity is associated with an increased inflammatory state, this route could be involved in the association between genetically determined PA and circulating immune cells [37]. Aside from the leading variants, the association with obesity-related traits was also observed in the MR-base look-up with the other genetic variants. However, further mechanistic studies are warranted to provide more insights into these possible additional pathways. 

A major strength of our study is the large cohort size of 222,645 participants. Second, this is the first study to assess genetic variants of PA using SNPs that were found in a GWAS performed with objectively measured PA data using wrist-worn accelerometer data. Thirdly, we used strict exclusion criteria to confine factors affecting immune cells such as infections. Furthermore, we used a stringent threshold *p*-value to reduce false positive rates and increase reproducibility. 

As a future perspective, it would be of additional value to investigate the association with the functionality and activity of cells, for example by examining circulating cytokine levels. Circulating cytokines were not measured in the UK Biobank and therefore not tested in the present study. This study could, however, serve as a starting point, providing new insights into where to focus future studies on the association between PA and circulating blood cells.

## 5. Conclusions

In conclusion, this study shows that genetically determined PA is associated with changes in circulating blood cells. Increased genetically determined PA is associated with decreased lymphocyte and eosinophil levels. Although further mechanistic studies are warranted, these findings cautiously suggest lifestyle changes that include more PA should be encouraged to improve the inflammatory state.

## Figures and Tables

**Table 1:** Baseline characteristics.

Characteristic	No. (%)
Total, No	222,645
Age, mean (SD), years	56 (8)
Sex, male (%)	105,970 (47.6%)
Body Mass Index, mean (SD), kg/m^2^	27.0 (4.4)
Smoking behavior, No (%)	
Never or <100 cigarettes	125,777 (58.1%)
Ex-smokers	66,895 (30.9%)
Current	23,849 (11.0%)
Hypertension, No (%)	60,900 (27.4%)
Hyperlipidemia, No (%)	40,099 (18.0%)
Diabetes Mellitus type 2, No (%)	6,984 (3.1%)
**PA phenotypes**	
Moderate PA, median (IQR), h/week	4.9 (1.5–11.3)
Vigorous PA, median (IQR), h/week	0.8 (0.0–2.7)
**Blood cell counts**	
Leukocytes, median (IQR), 10^9^/L	6.57 (5.60–7.70)
Erythrocytes, median (IQR), 10^12^/L	4.54 (4.29–4.82)
Neutrophils, median (IQR), 10^9^/L	3.95 (3.20–4.82)
Lymphocytes, median (IQR), 10^9^/L	0.92 (0.60–1.20)
Monocytes, median (IQR), 10^9^/L	0.44 (0.36–0.55)
Eosinophils, median (IQR), 10^9^/L	0.13 (0.10–0.21)
Basophils, median (IQR), 10^9^/L	0.02 (0.00–0.04)
Reticulocytes, median (IQR), 10^12^/L	0.06 (0.04–0.07)
Thrombocytes, median (IQR), 10^9^/L	246.8 (212.9–285.0)

Data is shown as number (%), as mean with standard deviation (SD) or as median with inter-quartile range (IQR). Units of measurements are indicated. PA: physical activity.

**Table 2:** Associations between increased physical activity and circulating blood cells.

	N snps	Inverse Variance Weighted (Fixed Effects)			Inverse Variance Weighted (Multiplicative Random Effects)			MR Egger Fixed Effects			Weighted Median		
		Beta	SE	*p*-Value	Beta	SE	*p*-Value	Beta	SE	*p*-Value	Beta	SE	*p*-Value
Leukocyte count (10^^9^ cells/L)	68	–0.0406	0.0307	1.86 × 10^−1^	–0.0406	0.0465	3.83 × 10^−1^	0.0106	0.1455	9.42 × 10^−1^	0.0024	0.0487	9.61 × 10^−1^
Erythrocyte count (10^^12^ cells/L)	68	0.0001	0.0063	9.85 × 10^−1^	0.0001	0.0137	9.93 × 10^−1^	0.0811	0.0417	5.64 × 10^−2^	−0.0126	0.0098	1.99 × 10^−1^
Erythrocyte distribution width (%)	68	0.0078	0.0166	6.40 × 10^−1^	0.0078	0.0281	7.82 × 10^−1^	0.0562	0.0878	5.25 × 10^−1^	−0.0047	0.0251	8.53 × 10^−1^
Neutrophil count (10^^9^ cells/L)	68	0.0055	0.0243	8.20 × 10^−1^	0.0055	0.0357	8.77 × 10^−1^	0.0768	0.1118	4.95 × 10^−1^	−0.0099	0.0369	7.88 × 10^−1^
Neutrophils (%)	68	0.4236	0.1502	4.80 × 10^−3^	0.4236	0.2288	6.41 × 10^−2^	0.8990	0.7164	2.14 × 10^−1^	0.0012	0.2359	9.96 × 10^−1^
Lymphocyte count (10^^9^ cells/L)	68	–0.0259	0.0081	1.40 × 10^−3^	–0.0259	0.0144	7.21 × 10^−2^	–0.0317	0.0454	4.87 × 10^−1^	−0.0305	0.0134	2.24 × 10^-2^
Lymphocyte (%)	68	–0.3606	0.1294	5.30 × 10^−3^	–0.3606	0.2016	7.37 × 10^−2^	–0.8313	0.6300	1.92 × 10^−1^	0.0836	0.1995	6.75 × 10^−1^
Monocyte count (10^^9^ cells/L)	68	–0.0010	0.0035	7.66 × 10^−1^	–0.0010	0.0045	8.21 × 10^−1^	0.0057	0.0140	6.86 × 10^−1^	−0.0013	0.0052	8.09 × 10^−1^
Monocytes (%)	68	0.0158	0.0462	7.33 × 10^−1^	0.0158	0.0613	7.97 × 10^−1^	0.0549	0.1919	7.76 × 10^−1^	0.0116	0.0739	8.75 × 10^−1^
Eosinophil count (10^^9^ cells/L)	68	–0.0078	0.0024	1.40 × 10^−3^	–0.0078	0.0039	4.90 × 10^−2^	–0.0071	0.0123	5.67 × 10^−1^	−0.0036	0.0036	3.23 × 10^−1^
Eosinophils (%)	68	–0.0687	0.0340	4.36 × 10^−2^	–0.0687	0.0619	2.67 × 10^−1^	–0.1215	0.1946	5.35 × 10^−1^	0.0021	0.0534	9.69 × 10^−1^
Basophil count (10^^9^ cells/L)	68	–0.0010	0.0009	2.89 × 10^−1^	–0.0010	0.0009	2.71 × 10^−1^	–0.0006	0.0028	8.32 × 10^−1^	−0.0027	0.0013	4.54 × 10^−2^
Basophils (%)	68	–0.0141	0.0110	1.99 × 10^−1^	–0.0141	0.0110	1.99 × 10^−1^	–0.0131	0.0332	6.94 × 10^−1^	−0.0262	0.0161	1.04 × 10^−1^
Reticulocyte count (10^^12^ cells/L)	68	–0.0256	0.0111	2.04 × 10^−2^	–0.0256	0.0188	1.73 × 10^−1^	0.0260	0.0586	6.59 × 10^−1^	−0.0455	0.0173	8.40 × 10^−3^
Reticulocytes (%)	68	–0.0242	0.0108	2.50 × 10^−2^	–0.0242	0.0165	1.41 × 10^−1^	–0.0001	0.0517	9.99 × 10^−1^	−0.0479	0.0161	3.00 × 10^−3^
Reticulocyte volume (femtolitres)	68	–0.3151	0.1397	2.41 × 10^−2^	–0.3151	0.2491	2.06 × 10^−1^	–0.9755	0.7778	2.14 × 10^−1^	0.0022	0.2097	9.92 × 10^−1^
Immature reticuloyctes fraction	68	–0.0027	0.0011	1.42 × 10^-2^	–0.0027	0.0016	1.01 × 10^−1^	0.0026	0.0051	6.15 × 10^−1^	−0.0055	0.0017	1.30 × 10^−3^
Platelet count (10^^9^ cells/L)	68	–2.5671	1.0319	1.29 × 10^−2^	–2.5671	1.8209	1.59 × 10^−1^	2.7242	5.6655	6.32 × 10^−1^	1.4266	1.6611	3.90 × 10^−1^
Platelet volume (femtolitres)	68	–0.0001	0.0199	9.96 × 10^−1^	–0.0001	0.0352	9.98 × 10^−1^	–0.1221	0.1092	2.68 × 10^−1^	0.0083	0.0334	8.04 × 10^−1^
Platelet packed cell volume (%)	68	–0.0025	0.0008	2.50 × 10^−3^	–0.0025	0.0014	6.65 × 10^−2^	–0.0003	0.0043	9.41 × 10^−1^	0.0000	0.0013	9.76 × 10^−1^
Platelet distribution width (%)	68	0.0369	0.0094	1.00 × 10^−4^	0.0369	0.0146	1.14 × 10^−2^	–0.0499	0.0443	2.64 × 10^−1^	0.0106	0.0144	4.64 × 10^−1^
Hemoglobin (g/dL)	68	0.0009	0.0177	9.60 × 10^−1^	0.0009	0.0315	9.78 × 10^−1^	0.1597	0.0974	1.06 × 10^−1^	−0.0216	0.0274	4.30 × 10^−1^
Hematocrit (%)	68	–0.0500	0.0521	3.37 × 10^−1^	–0.0500	0.0948	5.98 × 10^−1^	0.5543	0.2887	5.92 × 10^−2^	−0.0150	0.0816	8.54 × 10^−1^
Mean corpuscular volume (femtoliters)	68	–0.1090	0.0783	1.64 × 10^−1^	–0.1090	0.1126	3.33 × 10^−1^	–0.3913	0.3527	2.71 × 10^−1^	0.0492	0.1203	6.83 × 10^−1^
Mean corpuscular hemoglobin (picograms)	68	–0.0078	0.0321	8.08 × 10^−1^	–0.0078	0.0447	8.61 × 10^−1^	–0.2389	0.1367	8.52 × 10^−2^	0.0500	0.0488	3.05 × 10^−1^
Mean corpuscular hemoglobin concentration (grams/dL)	68	0.0402	0.0187	3.19 × 10^−2^	0.0402	0.0237	8.90 × 10^−2^	–0.0970	0.0719	1.82 × 10^−1^	0.0304	0.0284	2.84 × 10^−1^
Mean sphered cells volume (femtoliters)	68	–0.0564	0.0955	5.55 × 10^−1^	–0.0564	0.1836	7.59 × 10^−1^	–0.5939	0.5733	3.04 × 10^−1^	0.2760	0.1451	5.72 × 10^−2^

## Supplementary Materials

The following are available online at https://www.mdpi.com/2073-4425/10/11/908/s1, Supplementary Table S1: definitions of phenotypes UK Biobank; Supplementary Table S2: Single nucleotide polymorphisms previously associated with physical activity; Supplementary Table S3: Estimates of the associations between the single nucleotide polymorphisms and circulating blood cells; Supplementary Table S4: Mendelian randomization sensitivity analyses; Supplementary Table S5: MR-base look-up of associations between the 68 physical activity SNPs and other traits beside circulating immune cells, observed in the UK Biobank; Supplementary Figure S1: Scatter plot of physical activity single nucleotide polymorphisms on lymphocyte levels; Supplementary Figure S2: Scatter plot of physical activity single nucleotide polymorphisms on platelet width; Supplementary Figure S3: Scatter plot of physical activity single nucleotide polymorphisms on eosinophil level.
