# Supplementary material for: Genetically Determined Physical Activity and Its Association with Circulating Blood Cells
Source: Genes (Basel). 2019 Nov 7;10(11):908. doi: 10.3390/genes10110908 (PMC6895919; doi:10.3390/genes10110908)
Supplement: Supplementary file 1 [file genes-10-00908-s001.zip › Supplementary Figures.docx]

**Supplementary Figure 1.** Scatter plot of physical activity single nucleotide polymorphisms on lymphocyte levels.

**
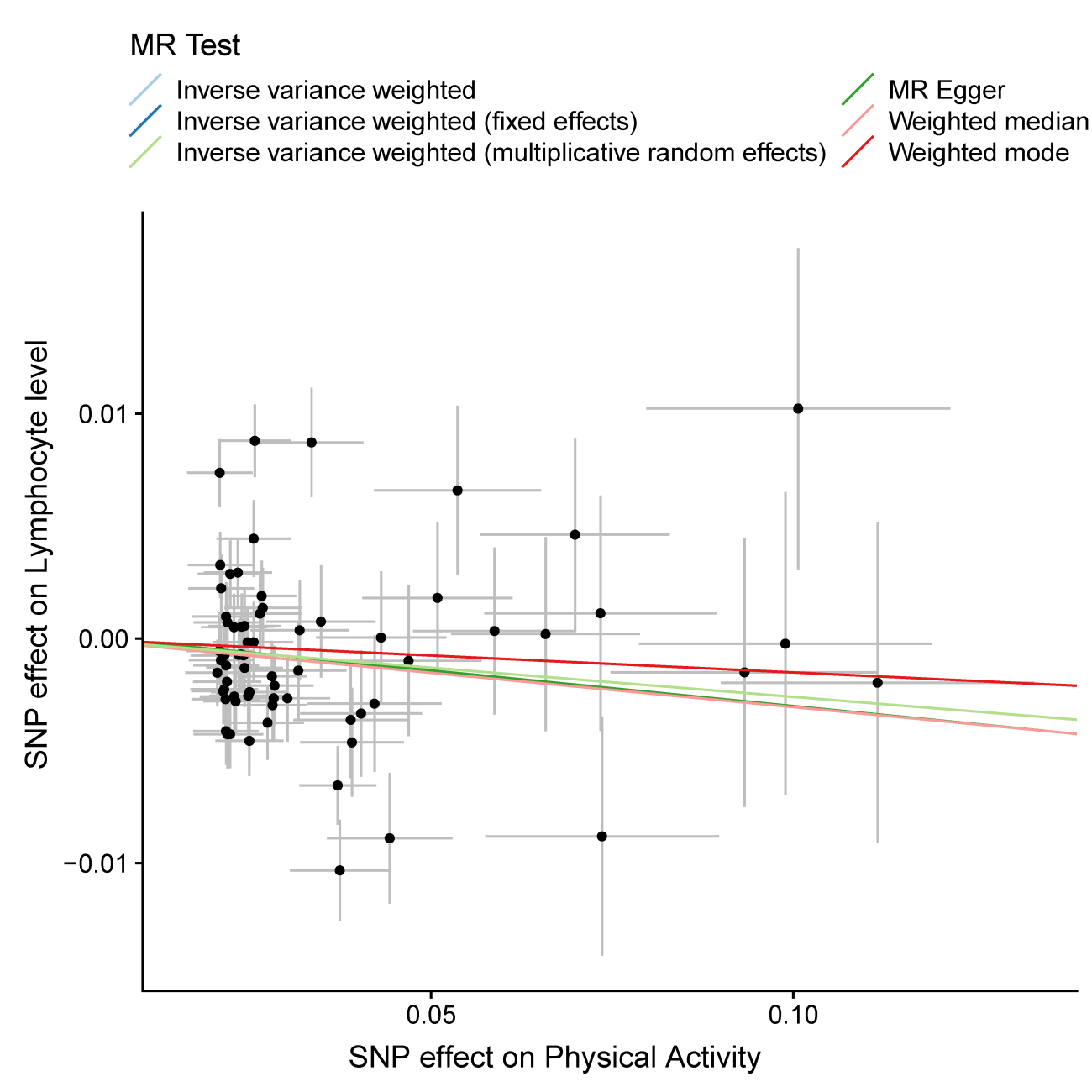
**

**Supplementary Figure 2.** Scatter plot of physical activity single nucleotide polymorphisms on platelet width.

**
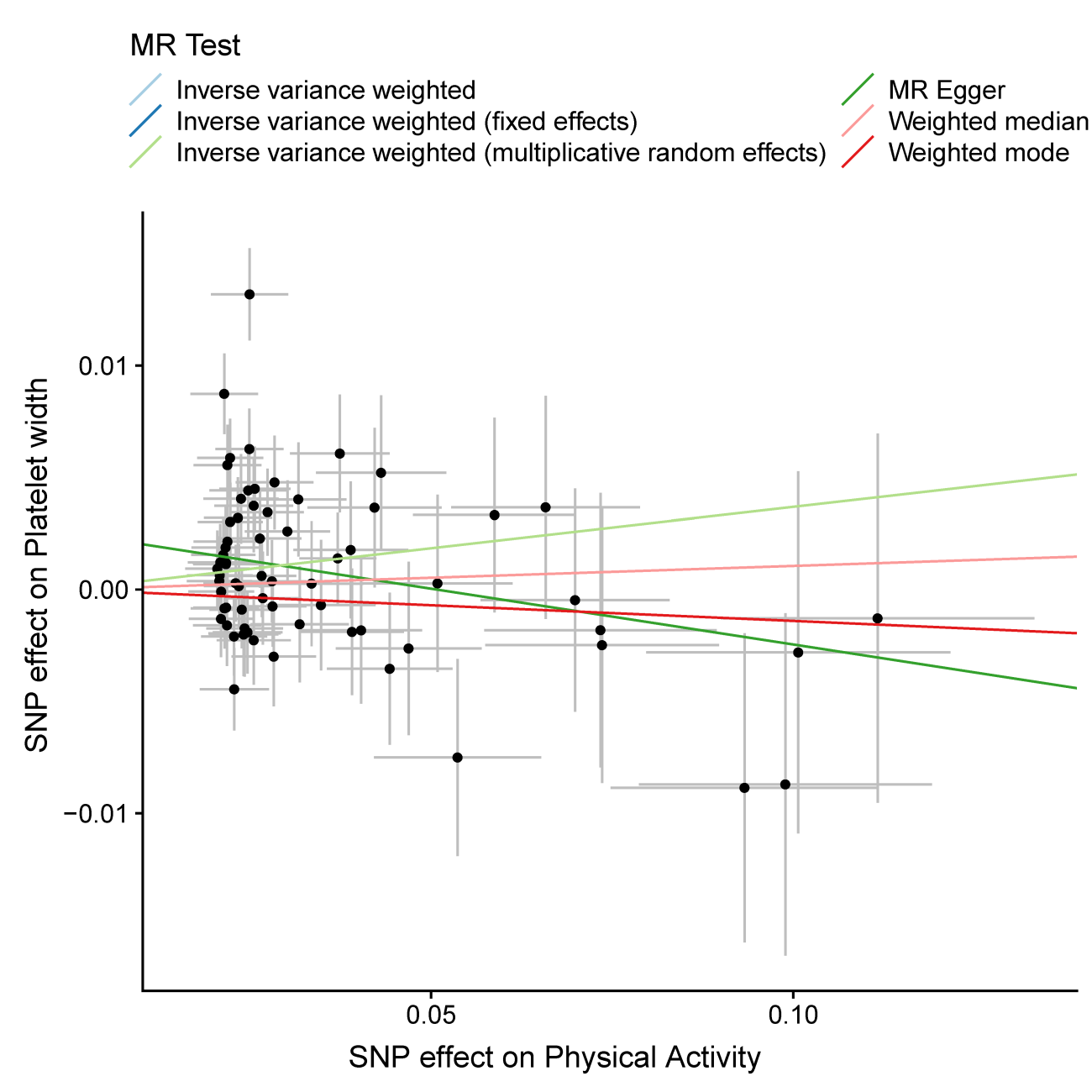
**

**Supplementary Figure 3.** Scatter plot of physical activity single nucleotide polymorphisms on eosinophil level.

**
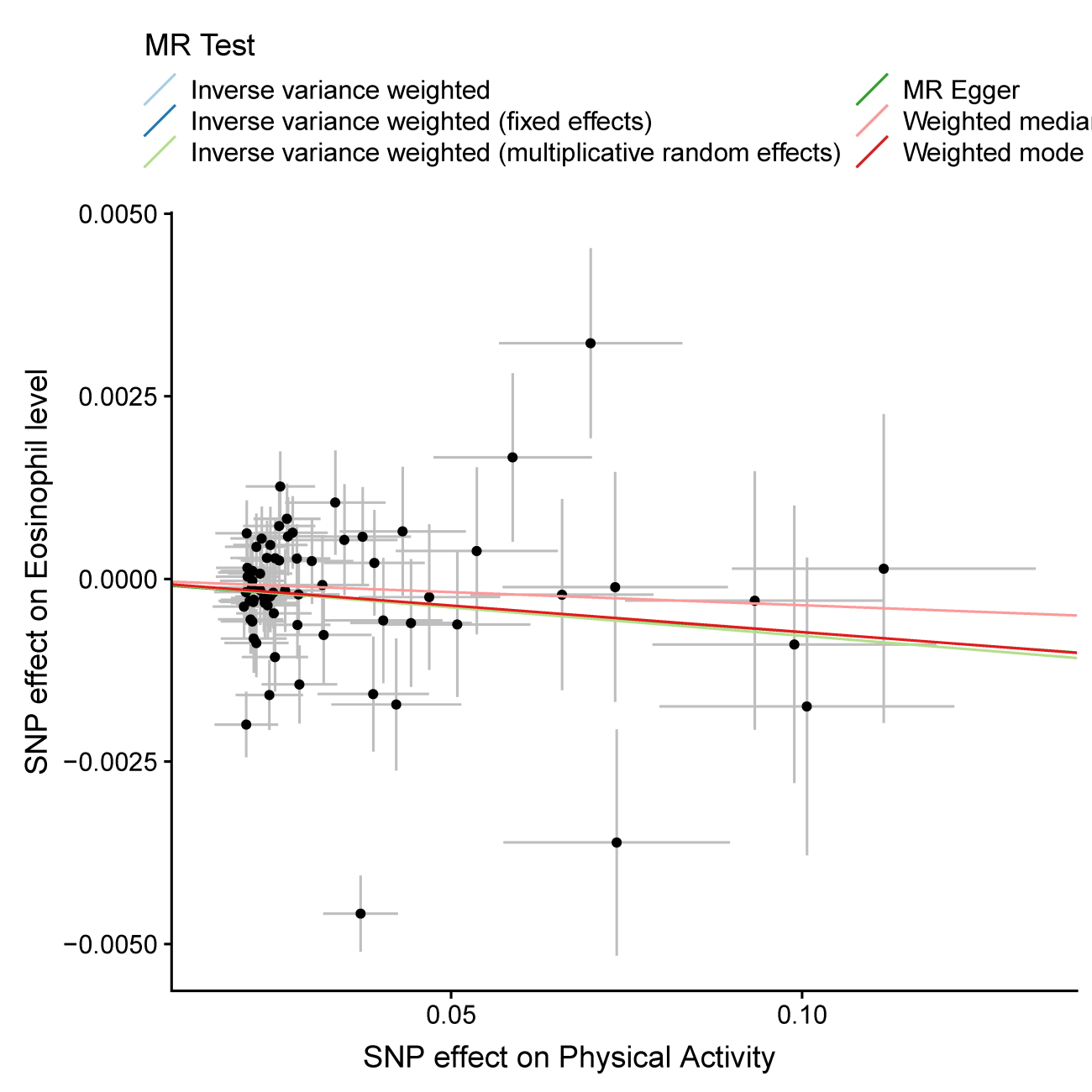
**
