# Supplementary material for: Genetically Determined Physical Activity and Its Association with Circulating Blood Cells
Source: Genes (Basel). 2019 Nov 7;10(11):908. doi: 10.3390/genes10110908 (PMC6895919; doi:10.3390/genes10110908)
Supplement: Supplementary file 1 [file genes-10-00908-s001.zip › Supplementary table 1- table 2 -table 4.docx]

Supplementary Table 1. Definitions phenotypes UK Biobank.

| **Variable** | **ICD-9** | **ICD-10** | **OPCS-4** | **Self-reported fields** | **Read codes** |
| --- | --- | --- | --- | --- | --- |
| Cancer (malignant) | 141, 142, 144, 146, 150, 151, 152, 153, 154, 155, 156, 157, 159, 160, 161, 162, 164, 170, 171, 172, 173, 174, 179, 180, 182, 183, 184, 185, 186, 187, 188, 189, 190, 191, 193, 195, 196, 198, 199, 200, 201, 202, 204, 205, V10 (Personal history of malignant neoplasm) | C, Z08 (Follow-up after treatment for malignant neoplasm), Z85 (Personal history of malignant neoplasm) |  | n_2453_= 1, n_20001_≥ 1 |  |
| Gout | 274 (Gout) | M10 (Gout) |  | 20002 (1466 (gout)) | j62, j61, j63, j64 |
| Inherited blood cell disorder | 282 (Hereditary haemolytic anaemias) | D55 (Anaemia due to enzyme disorders), D56 (Thalassaemia), D57 (Sickle-cell disorders), D58 (Other hereditary haemolytic anaemias) |  | 20002 (1339 (sickle cell disease), 1340 (thalassaemia)) |  |
| Pneumonia | 480, 481, 482, 483, 485, 486 | J100 (Influenza with pneumonia, virus identified), J110 (Influenza with pneumonia, virus not identified), J12 (Viral pneumonia, not elsewhere classified), J13 (Pneumonia due to Streptococcus pneumoniae), J14 (Pneumonia due to Haemophilus influenzae), J15 (Bacterial pneumonia, not elsewhere classified), J16 (Pneumonia due to other infectious organisms, not elsewhere classified), J17 (Pneumonia in diseases classified elsewhere), J18 (Pneumonia, organism unspecified) |  | 20002 (1398 (pneumonia)) |  |
| Rheumatoid arthritis | 714.0 (Rheumatoid arthritis) | M053(Rheumatoid arthritis with involvement of other organs and systems), M058 (Other seropositive rheumatoid arthritis), M059 (Seropositive rheumatoid arthritis, unspecified), M06 (Other rheumatoid arthritis), M068 (Other specified rheumatoid arthritis), and M069(Rheumatoid arthritis, unspecified) |  | 20002 (1464 (rheumatoid arthritis)) |  |
| Rheumatic heart disease | 391(Rheumatic fever with heart involvement), 394, 395, 396, and 398 (Chronic rheumatic heart disease) | I01 (Rheumatic fever with heart involvement), I05 (Rheumatic mitral valve diseases), I06 (Rheumatic aortic valve diseases), I07 (Rheumatic tricuspid valve diseases), I08(Multiple valve diseases), and I09(Other rheumatic heart diseases) |  | 20002 (1479 (rheumatic fever)) |  |
| Urinary tract infection | 5990 | N390 |  | 20002 (1196(Urinary tract infection)) |  |

**Supplementary Table 2.** Single nucleotide polymorphisms previously associated with physical activity.

| **rsID** | **Chr** | **hg19 position** | **EFAL** | **NEFAL** | **EF Frequency** | **Beta** | **SE** | ***P* value** |
| --- | --- | --- | --- | --- | --- | --- | --- | --- |
| rs159963 | 1 | 8504421 | C | A | 0.4225 | 0.0208 | 0.0045 | 4.40×10^-6^ |
| rs78681635 | 1 | 14654840 | C | A | 0.9804 | -0.0736 | 0.0161 | 4.90×10^-6^ |
| rs16835316 | 1 | 33691065 | C | G | 0.7830 | -0.0284 | 0.0054 | 1.20×10^-7^ |
| rs34517439 | 1 | 78450517 | C | A | 0.8791 | 0.0374 | 0.0069 | 5.10×10^-8^ |
| rs111205056 | 1 | 84358066 | T | C | 0.9624 | 0.0658 | 0.0130 | 4.10×10^-7^ |
| rs791273 | 1 | 147234618 | A | G | 0.7482 | -0.0255 | 0.0051 | 6.20×10^-7^ |
| rs76223101 | 2 | 118974070 | G | A | 0.9486 | -0.0509 | 0.0104 | 9.30×10^-7^ |
| rs12989603 | 2 | 152216827 | A | C | 0.6984 | 0.0235 | 0.0048 | 1.00×10^-6^ |
| rs1220114 | 2 | 158543700 | T | A | 0.7426 | 0.0238 | 0.0052 | 4.70×10^-6^ |
| rs7557793 | 2 | 171665325 | T | C | 0.7785 | -0.0248 | 0.0054 | 3.90×10^-6^ |
| rs7591144 | 2 | 221075004 | G | A | 0.6128 | -0.0210 | 0.0045 | 3.40×10^-6^ |
| rs4040769 | 3 | 10434490 | G | A | 0.6371 | -0.0217 | 0.0046 | 2.70×10^-6^ |
| rs6775319 | 3 | 18758501 | A | T | 0.2707 | 0.0274 | 0.0050 | 3.90×10^-8^ |
| rs16860042 | 3 | 147755033 | T | C | 0.9036 | -0.0348 | 0.0075 | 3.80×10^-6^ |
| rs651165 | 3 | 172412887 | G | A | 0.7700 | 0.0243 | 0.0053 | 4.40×10^-6^ |
| rs7626095 | 3 | 176320312 | C | T | 0.5624 | -0.0217 | 0.0045 | 1.70×10^-6^ |
| rs7658462 | 4 | 3283422 | C | T | 0.8284 | 0.0302 | 0.0059 | 3.30×10^-7^ |
| rs111789382 | 4 | 6746242 | T | C | 0.9339 | -0.0422 | 0.0093 | 4.80×10^-6^ |
| rs147350938 | 4 | 9924493 | C | T | 0.9869 | -0.0989 | 0.0202 | 1.10×10^-6^ |
| rs73249344 | 4 | 19543063 | A | G | 0.8139 | -0.0264 | 0.0057 | 4.50×10^-6^ |
| rs75321673 | 4 | 113558874 | A | G | 0.9594 | -0.0537 | 0.0115 | 3.70×10^-6^ |
| rs115354001 | 4 | 159474319 | C | T | 0.9824 | -0.0933 | 0.0185 | 4.80×10^-7^ |
| rs10475334 | 5 | 6572494 | G | A | 0.8716 | 0.0317 | 0.0066 | 1.90×10^-6^ |
| rs77296172 | 5 | 87519964 | G | A | 0.9182 | 0.0403 | 0.0084 | 1.70×10^-6^ |
| rs9293503 | 5 | 87948962 | T | C | 0.8884 | 0.0391 | 0.0072 | 5.00×10^-8^ |
| rs17135735 | 5 | 112917803 | C | T | 0.9292 | 0.0443 | 0.0087 | 3.00×10^-7^ |
| rs154103 | 5 | 151481997 | T | C | 0.3445 | -0.0233 | 0.0047 | 7.80×10^-7^ |
| rs6895232 | 5 | 152039421 | T | A | 0.6629 | 0.0266 | 0.0048 | 2.50×10^-8^ |
| rs945890 | 6 | 130321899 | A | T | 0.2872 | -0.0257 | 0.0049 | 1.90×10^-7^ |
| rs4314553 | 7 | 39304256 | T | C | 0.5161 | 0.0213 | 0.0044 | 1.60×10^-6^ |
| rs6945984 | 7 | 99348328 | T | C | 0.8926 | -0.0335 | 0.0072 | 3.00×10^-6^ |
| rs532259022 | 8 | 4856717 | A | C | 0.4315 | -0.0215 | 0.0047 | 4.20×10^-6^ |
| rs73715570 | 8 | 145025298 | C | T | 0.7703 | -0.0249 | 0.0053 | 2.70×10^-6^ |
| rs2821176 | 9 | 11642328 | T | G | 0.7280 | 0.0242 | 0.0050 | 1.10×10^-6^ |
| rs10120942 | 9 | 87597283 | C | A | 0.4302 | 0.0209 | 0.0045 | 3.20×10^-6^ |
| rs1268539 | 9 | 128195657 | C | A | 0.5812 | -0.0223 | 0.0045 | 7.20×10^-7^ |
| rs564819152 | 10 | 21820650 | A | G | 0.6788 | 0.0280 | 0.0048 | 4.20×10^-9^ |
| rs11007336 | 10 | 29276450 | G | A | 0.5367 | 0.0208 | 0.0045 | 3.80×10^-6^ |
| rs9300002 | 11 | 29129168 | T | C | 0.5790 | -0.0217 | 0.0045 | 1.40×10^-6^ |
| rs148193266 | 11 | 104528681 | A | C | 0.9573 | -0.0588 | 0.0113 | 1.80×10^-7^ |
| rs34207584 | 12 | 34663267 | G | C | 0.6447 | -0.0219 | 0.0047 | 2.90×10^-6^ |
| rs4002684 | 12 | 37896288 | A | T | 0.5965 | -0.0223 | 0.0046 | 1.00×10^-6^ |
| rs10880697 | 12 | 38594529 | G | C | 0.6609 | -0.0249 | 0.0047 | 1.20×10^-7^ |
| rs78982639 | 12 | 70604120 | G | A | 0.9886 | 0.1007 | 0.0210 | 1.60×10^-6^ |
| rs75972257 | 13 | 40508738 | C | A | 0.9116 | 0.0389 | 0.0079 | 9.60×10^-7^ |
| rs9529057 | 13 | 66984458 | G | A | 0.5265 | -0.0210 | 0.0044 | 2.30×10^-6^ |
| rs11157124 | 14 | 22151759 | A | G | 0.6627 | -0.0216 | 0.0047 | 4.80×10^-6^ |
| rs5742915 | 15 | 74336633 | T | C | 0.5393 | -0.0230 | 0.0045 | 2.40×10^-7^ |
| rs1105867 | 15 | 83439083 | T | C | 0.2241 | -0.0268 | 0.0053 | 5.40×10^-7^ |
| rs8033999 | 15 | 92169857 | T | C | 0.4698 | 0.0205 | 0.0044 | 4.20×10^-6^ |
| rs12448218 | 16 | 8705547 | G | A | 0.6606 | -0.0218 | 0.0047 | 3.20×10^-6^ |
| rs28529261 | 16 | 47197865 | T | C | 0.9862 | -0.1116 | 0.0216 | 2.50×10^-7^ |
| rs9938281 | 16 | 49625336 | A | G | 0.4740 | -0.0239 | 0.0045 | 9.60×10^-8^ |
| rs4640189 | 16 | 86952035 | C | T | 0.9341 | -0.0431 | 0.0090 | 1.60×10^-6^ |
| rs2696625 | 17 | 44326864 | A | G | 0.7704 | -0.0371 | 0.0053 | 3.20×10^-12^ |
| rs12449794 | 17 | 74880716 | C | T | 0.9456 | 0.0469 | 0.0101 | 3.20×10^-6^ |
| rs186056728 | 18 | 221643 | C | G | 0.9676 | -0.0699 | 0.0131 | 8.70×10^-8^ |
| rs1668835 | 18 | 22478952 | T | A | 0.6883 | -0.0247 | 0.0048 | 2.80×10^-7^ |
| rs59499656 | 18 | 40768309 | A | T | 0.6555 | -0.0281 | 0.0047 | 1.90×10^-9^ |
| rs28418831 | 19 | 3307260 | A | C | 0.6316 | -0.0219 | 0.0047 | 3.20×10^-6^ |
| rs62125156 | 19 | 7943575 | A | C | 0.7875 | 0.0255 | 0.0054 | 3.10×10^-6^ |
| rs12460611 | 19 | 30326600 | A | G | 0.6770 | 0.0228 | 0.0048 | 1.80×10^-6^ |
| rs157595 | 19 | 45425460 | A | G | 0.3851 | -0.0214 | 0.0047 | 4.20×10^-6^ |
| rs10415609 | 19 | 46822760 | A | G | 0.1222 | 0.0319 | 0.0068 | 2.60×10^-6^ |
| rs6081105 | 20 | 1886493 | A | G | 0.3988 | -0.0228 | 0.0045 | 5.10×10^-7^ |
| rs1124167 | 20 | 38541242 | G | C | 0.9798 | 0.0734 | 0.0160 | 4.70×10^-6^ |
| rs79980391 | 21 | 17319794 | T | A | 0.8200 | -0.0283 | 0.0058 | 1.20×10^-6^ |
| rs112779734 | 22 | 41434198 | T | C | 0.6922 | 0.0241 | 0.0048 | 5.40×10^-7^ |

Single nucleotide polymorphisms previously associated with physical activity by Doherty et al. Abbreviations: Chr, Chromosome; EFAL, Effect allele; NEFAL, Non-Effect Allele; EF Frequency, Effect Allele Frequency; SE, Standard Error

Supplementary Table 4. Mendelian Randomization sensitivity analyses

| **Outcome** | **MR-Egger intercept** | | | **Cochran's Q** | | | **Rucker’s Q** | | | **Q-Q'** | | |
| --- | --- | --- | --- | --- | --- | --- | --- | --- | --- | --- | --- | --- |
|  | **Intercept** | **SE** | **P value** | **Q** | **DF** | **P-value** | **Q** | **DF** | **P-value** | **Q-Q'** | **DF** | **P-value** |
| Hemoglobin | -0.0046 | 0.0027 | 8.99×10^-02^ | 210 | 67 | 4.30×10^-17^ | 200 | 66 | 5.70×10^-16^ | 9.1 | 1 | 2.50×10^-03^ |
| Hematocrit | -0.0176 | 0.0080 | 3.07×10^-02^ | 220 | 67 | 1.70×10^-18^ | 210 | 66 | 2.00×10^-16^ | 15.0 | 1 | 9.30×10^-05^ |
| Lymphocyte count | 0.0002 | 0.0013 | 8.94×10^-01^ | 210 | 67 | 4.60×10^-17^ | 210 | 66 | 2.60×10^-17^ | 0.1 | 1 | 8.10×10^-01^ |
| Lympocytes (%) | 0.0137 | 0.0174 | 4.33×10^-01^ | 160 | 67 | 6.50×10^-10^ | 160 | 66 | 6.50×10^-10^ | 1.5 | 1 | 2.20×10^-01^ |
| Mean corpuscular hemoglobin concentration | 0.0040 | 0.0020 | 4.80×10^-02^ | 107 | 67 | 1.50×10^-3^ | 100 | 66 | 4.00×10^-3^ | 6.2 | 1 | 1.29×10^-02^ |
| Mean corpuscular hemoglobin | 0.0067 | 0.0038 | 7.88×10^-02^ | 130 | 67 | 7.10×10^-06^ | 120 | 66 | 2.30×10^-05^ | 6.0 | 1 | 1.50×10^-02^ |
| Mean corpuscluar volume | 0.0082 | 0.0097 | 4.01×10^-01^ | 140 | 67 | 6.60×10^-07^ | 140 | 66 | 6.70×10^-07^ | 1.5 | 1 | 2.20×10^-01^ |
| Monocyte count | -0.0002 | 0.0004 | 6.14×10^-01^ | 120 | 67 | 2.30×10^-04^ | 110 | 66 | 1.90×10^-04^ | 0.5 | 1 | 5.00×10^-01^ |
| Monocytes (%) | -0.0011 | 0.0053 | 8.30×10^-01^ | 120 | 67 | 1.30×10^-04^ | 120 | 66 | 9.50×10^-05^ | 0.1 | 1 | 7.70×10^-01^ |
| Mean sphered cells volume | 0.0157 | 0.0158 | 3.26×10^-01^ | 250 | 67 | 1.70×10^-22^ | 240 | 66 | 3.40×10^-22^ | 3.6 | 1 | 5.70×10^-02^ |
| Neutrophil count | -0.0021 | 0.0031 | 5.03×10^-01^ | 150 | 67 | 9.90×10^-08^ | 140 | 66 | 8.70×10^-08^ | 1.0 | 1 | 3.20×10^-01^ |
| Neutrophils (%) | -0.0139 | 0.0198 | 4.86×10^-01^ | 160 | 67 | 5.50×10^-09^ | 150 | 66 | 5.00×10^-09^ | 1.1 | 1 | 2.80×10^-01^ |
| Platelet packed cell volume | -0.0001 | 0.0001 | 5.91×10^-01^ | 180 | 67 | 1.70×10^-12^ | 180 | 66 | 1.30×10^-12^ | 0.8 | 1 | 3.70×10^-01^ |
| Platelet count | -0.1545 | 0.1566 | 3.28×10^-01^ | 210 | 67 | 1.90×10^-16^ | 210 | 66 | 3.00×10^-16^ | 3.0 | 1 | 8.20×10^-02^ |
| Platelet volume | 0.0036 | 0.0030 | 2.42×10^-01^ | 210 | 67 | 1.10×10^-16^ | 210 | 66 | 2.80×10^-16^ | 4.3 | 1 | 3.70×10^-02^ |
| Platelet distribution width | 0.0025 | 0.0012 | 4.24×10^-02^ | 160 | 67 | 1.30×10^-09^ | 150 | 66 | 1.50×10^-08^ | 9.8 | 1 | 1.80×10^-03^ |
| Erythrocyte count | -0.0024 | 0.0012 | 4.47×10^-02^ | 320 | 67 | 7.40×10^-34^ | 300 | 66 | 5.80×10^-31^ | 19.0 | 1 | 1.40×10^-05^ |
| Erythrocyte distribution width | -0.0014 | 0.0024 | 5.63×10^-01^ | 190 | 67 | 7.00×10^-14^ | 190 | 66 | 5.60×10^-14^ | 1.0 | 1 | 3.20×10^-01^ |
| Immature reticuloyctes fraction | -0.0002 | 0.0001 | 2.80×10^-01^ | 150 | 67 | 3.30×10^-08^ | 150 | 66 | 4.60×10^-08^ | 2.6 | 1 | 1.00×10^-01^ |
| Reticulocyte count | -0.0015 | 0.0016 | 3.56×10^-01^ | 190 | 67 | 3.20×10^-14^ | 190 | 66 | 4.30×10^-14^ | 2.5 | 1 | 1.10×10^-01^ |
| Reticulocytes (%) | -0.0007 | 0.0014 | 6.24×10^-01^ | 160 | 67 | 5.70×10^-09^ | 150 | 66 | 4.40×10^-09^ | 0.6 | 1 | 4.50×10^-01^ |
| Reticulocyte volume | 0.0193 | 0.0215 | 3.73×10^-01^ | 210 | 67 | 3.90×10^-17^ | 210 | 66 | 5.20×10^-17^ | 2.6 | 1 | 1.10×10^-01^ |
| Leukocyte count | -0.0015 | 0.0040 | 7.12×10^-01^ | 150 | 67 | 8.70×10^-09^ | 150 | 66 | 6.10×10^-09^ | 0.3 | 1 | 5.70×10^-01^ |
| Eosinophil count | -0.00002 | 0.0003 | 9.54×10^-01^ | 180 | 67 | 6.10×10^-12^ | 180 | 66 | 3.70×10^-12^ | 0.0 | 1 | 9.20×10^-01^ |
| Basophil count | -0.00001 | 0.0001 | 8.85×10^-01^ | 62 | 67 | 6.49×10^-1^ | 62 | 66 | 6.16×10^-1^ | 0.0 | 1 | 8.85×10^-01^ |
| Eosinophils (%) | 0.0015 | 0.0054 | 7.75×10^-01^ | 220 | 67 | 2.20×10^-18^ | 220 | 66 | 1.30×10^-18^ | 0.3 | 1 | 6.00×10^-01^ |
| Basophils (%) | -0.00003 | 0.0009 | 9.74×10^-01^ | 67 | 67 | 4.78×10^-1^ | 70 | 66 | 4.43×10^-1^ | 0.0 | 1 | 9.74×10^-01^ |
